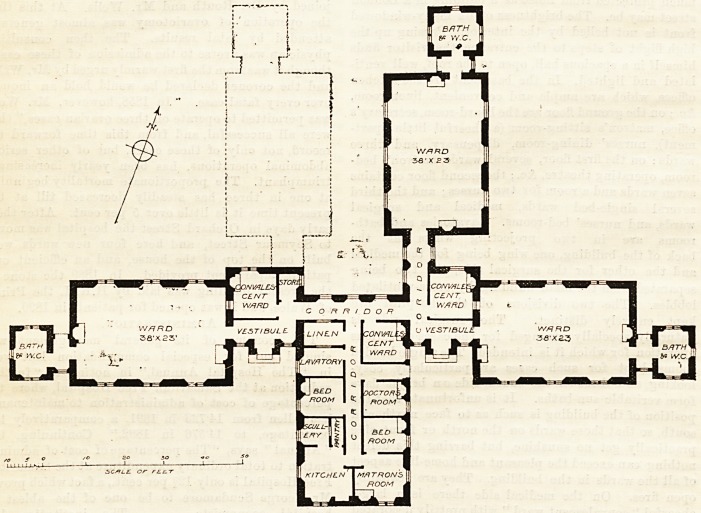# Hospital for Thurso District

**Published:** 1895-04-13

**Authors:** 


### HOSPITAL CONSTRUCTION.

### HOSPITAL FOR THURSO DISTRICT.

The plans with which we have been favoured would appear to have been submitted in competition, and are apparently for a hospital for infectious diseases. The whole of the buildings are on the ground floor, but it is suggested in the report that bed-rooms might in the future be added in an upper storey, though where it is proposed to place the staircase does not appear.

The central portion is occupied by administration offices, which consist of a matron's sitting-room, with her bed-room immediately adjoining, and approached only through the sitting-room. Adjoining the matron's sitting-room is the kitchen, the doors of these rooms being immediately opposite one another, and divided by a passage only four feet wide. Leading out of the kitchen is a narrow, dark, and unventilated cupboard, seven feet by three, called a pantry; and a scullery, six and a half feet by seven.

Next the matron's bed-room is a doctor's room, and next to that is the main entrance, only four feet wide. On the opposite side of the passage is a bed-room, ten feet by ten, which is apparently the only sleeping accommodation for the nursing staff night and day. Next to the last is a lavatory, and next to that a linen-room. On the left of the entrance is a convalescent room, very badly lighted, and about large enough for two patients.

Three main wards are suggested, each thirty-eight feet long and twenty-three feet wide, and each to hold six patients. The beds are not shown, but from the planning of the ward the only available spaces are in front of the windows! It is suggested in the report that each ward can be divided by a movable partition, and so made available for patients of both sexes. At the upper end of each ward is a room which appears to be designed for the purposes of a combined bath-room, lavatory, and water-closet. There are also two small rooms, eleven feet square, which are to be used as observation wards. No provision whatever is made for w.c.'s or bath-rooms, either for the matron and nurses or for the servants.

On the whole, it would be difficult to conceive a plan which sinned more than this against the rudiments of hospital construction.

## Figures and Tables

**Figure f1:**